# Prevalence and factors associated with unintended pregnancy among adolescent girls and young women in sub-Saharan Africa, a multilevel analysis

**DOI:** 10.1186/s12905-022-02048-7

**Published:** 2022-11-21

**Authors:** Hiwotie Getaneh Ayalew, Alemneh Mekuriaw Liyew, Zemenu Tadesse Tessema, Misganaw Gebrie Worku, Getayeneh Antehunegn Tesema, Tesfa Sewunet Alamneh, Achamyeleh Birhanu Teshale, Yigizie Yeshaw, Adugnaw Zeleke Alem

**Affiliations:** 1 Department of Midwifery, School of Nursing and Midwifery, College of Medicine and Health Sciences, Wollo University, Dessie, Ethiopia; 2 Department of Epidemiology and Biostatistics, Institute of Public Health, College of Medicine and Health Sciences and Comprehensive Specialized Hospital, University of Gondar, Gondar, Ethiopia; 3 Department of Human Anatomy, College of Medicine and Health Sciences and Comprehensive Specialized Hospital, University of Gondar, Gondar, Ethiopia; 4 Department of Human Physiology, College of Medicine and Health Sciences and Comprehensive Specialized Hospital, University of Gondar, Gondar, Ethiopia

**Keywords:** Prevalence, Unintended pregnancy, Adolescent girls and young women, Sub-Saharan Africa

## Abstract

**Background:**

Unintended pregnancy predisposes women to unsafe abortion, malnutrition, mental illness, and even death. Though adolescent girls and young women are at higher risk of unintended pregnancy, there is a paucity of evidence in its burden and associated factors in sub-Saharan Africa. Therefore, this study aimed to assess the prevalence and factors associated with unintended pregnancy among adolescent girls and young women in sub-Saharan Africa.

**Method:**

This study was a secondary data analysis of 36 sub-Saharan African countries with a total weighted sample of 17,797 adolescent girls and young women. A multilevel logistic regression model was fitted and, the Adjusted Odds Ratio (AOR) with a 95% Confidence Interval (CI) was reported to assess the association between the independent variables and unintended pregnancy in Sub-Saharan Africa.

**Result:**

The pooled prevalence of unintended pregnancy in sub-Saharan Africa was 30.01 with 95% CI (29.38–30.74). In multivariable multilevel logistic regression analysis, adolescent girls, and young women with higher education (AOR = 0.71 95%CI 0.52–0.97), those who know modern contraceptive methods (AOR = 0.86 95%CI 0.75–0.98), and traditional contraceptive methods (AOR = 0.90, 95%CI 0.59–0.95), married (AOR = 0.80, 95%CI 0.73–0.88), those from female-headed households (AOR = 0.86,95%CI 0.78–0.94), had lower odds of unintended pregnancy. Whereas adolescent girls and young women from Central Africa (AOR = 2.09,95%CI 1.23–3.55), southern Africa (AOR = 5.23, 95%CI 2.71–10.09), and Eastern Africa (AOR = 1.07,95%CI 1.07–2.66) had higher odds of unintended pregnancy.

**Conclusion:**

Prevalence of unintended pregnancy in Sub-Saharan Africa is high. Therefore, educating adolescent girls and young women, and improving their knowledge about family planning services is vital. It is also better for the government of countries in sub-Saharan Africa and other global and local stakeholders to work hard to ensure universal access to sexual and reproductive healthcare services, including family planning, education, and the integration of reproductive health into national strategies and programs to reduce unintended pregnancy.

## Background

Unintended pregnancy is a pregnancy that is mistimed, unplanned or unwanted at the time of conception [1]. It is usually an outcome of nonuse, inconsistent use, or incorrect use of effective family planning methods [1, 2]. A recent report from the United Nations sexual and reproductive health agency notified that globally, nearly half of all pregnancies are unintended [3]. Though there has been a reduction in unintended pregnancy globally, low- and middle-income countries are still victims of unintended pregnancy [4, 5]. While the global rate of unintended pregnancies in Europe and North America was 35 per 1000 women aged 15 to 49, in sub-Saharan Africa it was 91 per 1000 women [6]. Similarly, there was a substantial heterogeneity within in sub-Saharan African countries regarding unintended pregnancy [7].

Unintended pregnancy predisposes adolescent girls and young women to several risk factors such as unsafe abortion, maternal death, malnutrition, mental illness and vertical transmission of Human Immuno Virus (HIV) to children, and school dropout [8, 9]. Besides, in sub-Saharan Africa, unintended pregnancy predisposes about 1 in 16 women to psychosocial impacts of morbidity and mortality [10]. It also induces stress, affects women’s quality of life, and the economic status of families at large [11, 12].

In previous studies education [13–17], parity [13, 15, 16], place of residence [13, 18], wealth [13, 18], age [14, 15, 18], healthcare decision-making [14], sexual violence [19], knowledge of contraceptive methods [15–17], marital status [16–18, 20, 21], occupation [20], sex of household head [20], birth interval [20], region and family planning message [22] have a significant association with unintended pregnancy.

Though unintended pregnancy occurs at any age women, adolescent girls and young women (15–24 years) are at higher risk of unintended pregnancy [23]. Consequently, the risk of maternal mortality is likely to be higher among this age group as evidenced by pieces of literature [24, 25]. Moreover, women in this age category have higher fertility, higher frequency of sexual intercourse, lower knowledge of contraceptive methods, and higher rates of contraceptive failure relative to older women [26–29]. On the other hand, recent evidence has shown the inequalities and uneven progress in many key sexual health indicators within sub-Saharan Africa which makes adolescent girls and young women to be highly vulnerable to poor sexual health outcomes [30].

Even though they are at higher risk of unintended pregnancy and related complications, there is a paucity of information on the magnitude and its sociodemographic correlates in sub-Saharan Africa. Outside of sexual and reproductive health issues affecting adolescent girls and young women, limited research has been conducted on the broader context of adolescent health in SSA. Thus, a strengthened capacity to generate rigorous scientific evidence to inform policies and programs designed to improve young women’s health is needed. Therefore, this study is aimed to assess the prevalence and factors associated with unintended pregnancy in sub-Saharan Africa among adolescent girls and young women by using the most recent Demographic and health survey data from 36 countries.

## Methods

### Data source

The dataset for this study was obtained from the measure DHS program after permission was granted at http://www.dhsprogram.com. A total of 36 sub-Saharan African countries’ most recent DHS datasets from 2006 to 2019 were used in this study.

Data from the southern region of Africa (Lesotho, Namibia, Swaziland, and South Africa), the central region of Africa (Angola, Democratic Republic Congo, Congo, Cameroon, Chad, Gabon, Sao Tome & Principe), the Eastern region of Africa (Burundi, Comoros, Ethiopia, Kenya, Madagascar, Malawi, Mozambique, Rwanda, Tanzania, Uganda, Zambia, and Zimbabwe), western Africa (Burkina-Faso, Benin, Cote d’Ivoire, Ghana, Gambia, Guinea, Liberia, Mali, Nigeria, Niger, Sierra Leone, Senegal, and Togo) was included.

Each country’s survey consists of different datasets including men, women, children, birth, and household datasets. For this study, we used the individual records data set (IR file) where data on women’s health is recorded. Demographic and health survey is conducted at the five-year interval and follows a common execution procedure in each country.

A two-stage stratified sampling procedure is adopted to select study participants in each survey. In the first phase, Enumeration Areas (EAs) were selected based on the sampling frame of each respective country. In the second stage, a sample of households is drawn from each EA. Then eligible study participants were interviewed in the selected household. The detail of the sampling procedure has been documented elsewhere [31]. For the current, study a total of 17,797 (weighted sample) adolescent girls and young women [15–24] having a pregnancy at the time of the interview in 36 sub-Saharan African countries were included (Table 1). During analysis sampling weight was applied using individual sample weights recorded in the data set to produce reliable estimates by adjusting the over and under-sampled regions.

**Table 1:** Study participants included in the study from four sub-Saharan regions of Africa, 2006–2019

Region of Africa	Weighted Sample size	Percent (%)
Central Region of Africa	4267	23.98
Southern Region of Africa	706	3.97
Eastern Region of Africa	6250	35.12
Western Region of Africa	6574	36.94
Total	**17,797**	**100**

### Study variables

#### Dependent variable

The dependent variable for this study was unintended pregnancy. It was measured in such a way, by asking women about their pregnancy to state just when they wanted their pregnancy (then, later, or not at all). Those women responding to the above question as ‘wanted later’ or ‘not wanted at all were considered to have an unintended pregnancy and those who responded by saying ‘wanted then’ were considered to have intended pregnancy. Therefore, unintended pregnancy was coded ‘1’, and intended pregnancy was coded ‘0’ for further statistical analysis.

#### Independent variables

Individual and community-level variables were retrieved from DHS datasets. Age [15–24], educational level (no education, primary, secondary, and higher), marital status(single, married,), wealth index (Poorest, Poorer, poor, Richer, and Richest), media exposure (yes, no), heard about family planning from media (yes, no), knowledge of contraceptive methods (no, traditional, modern), distance to health facility (big problem and not a big problem), smoking (yes, no), covered by health insurance (no, yes), sex of household head (male, female) and occupation (not working, working) were individual-level variables. Whereas residence (urban and rural) and SSA region (South Africa, Central Africa, East Africa, and West Africa) were community (country) level variables.

### Statistical analysis

The descriptive statistics was presented in Table 2. The overall prevalence of unintended pregnancy among adolescent girls and young women in sub-Saharan Africa with 95%CI was reported. A multilevel logistic regression model was fitted to assess the factors associated with unintended pregnancy. Consequently, four models were fitted. First, the null model without explanatory variables was fitted by using the country as a group variable to assess the community (country) level variance and the applicability of multilevel analysis. Model II and model III were adjusted for individual-level variables and community-level variables respectively. In model IV, both individual-level and community-level variables were fitted simultaneously. Deviance was used for model comparison. Accordingly, the final (Model IV) was the best-fitted model. In bi-variable analysis variables which are eligible for multivariable analysis were selected at a *p*-value of 0.2. The multi-collinearity was checked using the variance inflation factor (VIF) to avoid the inflation of the effect size of independent variables. In the multivariable analysis, an Adjusted Odds Ratio (AOR) with 95% CI was reported and variables with a *p*-value ≤0.05 were considered significant determinants of unintended pregnancy.

**Table 2:** Sociodemographic characteristics of study participants in sub-Saharan Africa 2006–2019

Variables	Weighted Frequency	Percent
Age		
15–19	6821	38.33
20–24	10,976	61.67
Educational level		
No education	5627	31.62
Primary	6597	37.07
Secondary	5302	29.79
Higher	271	1.52
Sex of household head		
Male	14,279	80.23
Female	3518	19.77
Marital status		
Single	3167	17.80
Married	14,630	82.20
Wealth status		
Poorest	4349	24.44
Poorer	4125	23.18
Middle	3614	20.31
Richer	3233	18.17
Richest	2476	13.31
Media exposure		
No	6921	38.89
Yes	10,876	61.11
Knowledge of contraceptive methods		
No	1480	8.32
Traditional	113	0.63
Modern	16,204	91.05
Smoking		
No	15,956	89.66
Yes	1841	10.34
Distance from the health facility		60.96
Not a big problem	10,849	55.82
Big problem	6948	39.04
Heard family planning from media		
No	11,684	65.65
Yes	6113	34.35
Covered by health insurance		
No	15,411	86.59
Yes	2386	13.41
Occupation		
Not working	9198	51.68
Working	8599	48.32
Residence		
Urban	5249	29.49
Rural	12,548	70.51
SSA Region		
Southern Africa	706	3.97
Central Africa	4267	23.98
East Africa	6250	35.12
West Africa	6574	36.94
Total	17,797	100.00

## Result

A total of 17,797 adolescent girls and young women from 36 sub-Saharan African countries were included in this study. The median age of study participants was 20 years with an interquartile range of 4. The majority (70.51%) of the study participants were rural residents. About 31.62% of them had no formal education and above three-fourths of (80.20%) of study, participants were married. Nearly 24% of study participants were from households with the poorest wealth category. About 91% of study participants knew the modern contraceptive method and two-thirds (65.65) of study participants didn’t hear about family planning from the media. Regarding the region of residence majority (36.94) of respondents were from the western part of the Sub-Saharan African region (Table 2).

### The pooled prevalence of unintended pregnancy

**Fig. 1:**
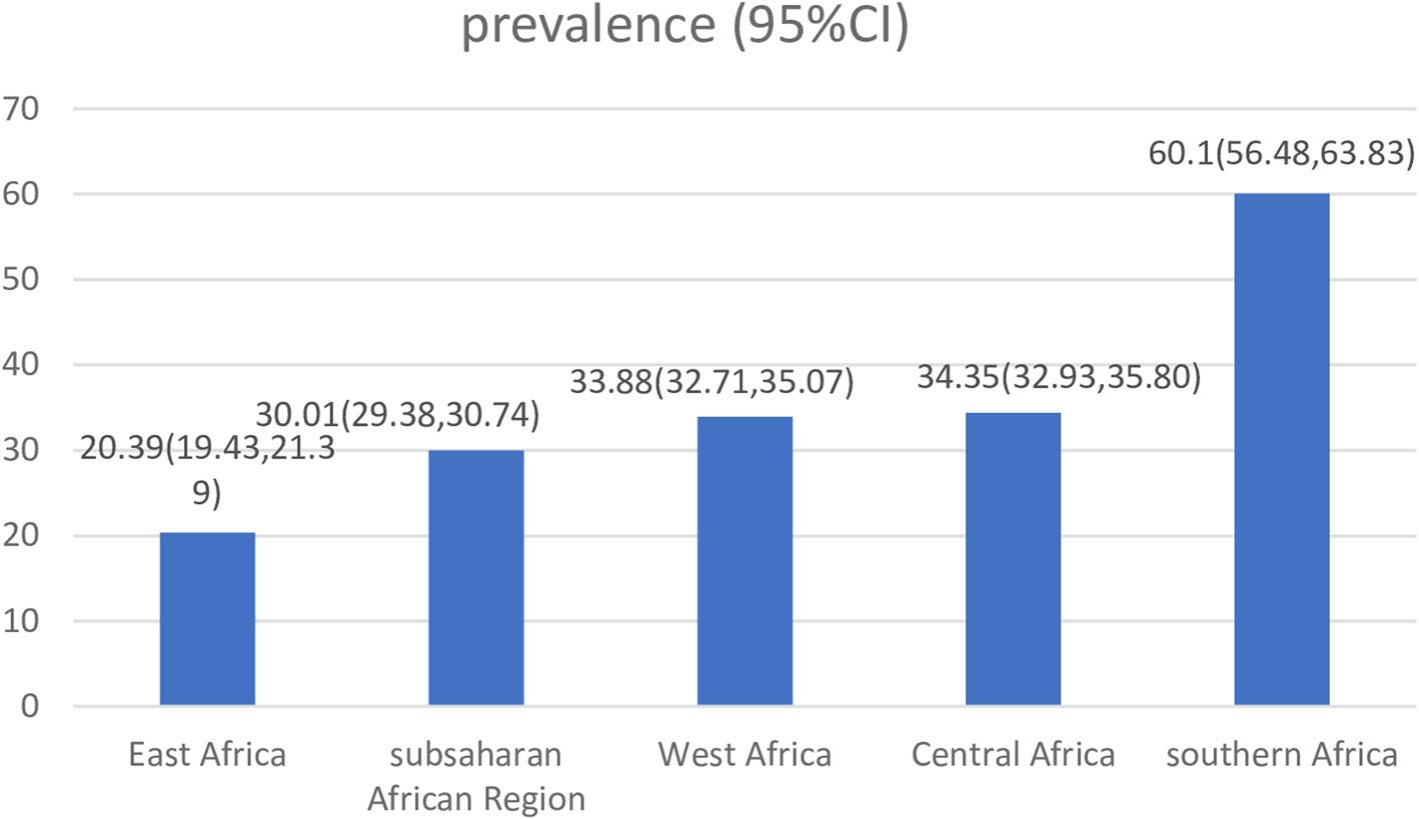
Prevalence of unintended pregnancy in sub-Saharan Africa 2006–2019

The overall prevalence of unintended pregnancy among adolescent girls and young women in sub-Saharan Africa was 30.01 with a 95% CI (29.38–30.74). The highest magnitude was observed in Southern Africa (60.01%) and the lowest in Eastern Africa (20.39%) region (Fig. 1).

### Random parameter estimation

The null model indicated that there was a significant clustering of unintended pregnancy across all 36 countries SSA (country/community variance =0.56, *p*-vale< 0.001). The intracluster correlation coefficient (ICC) ranges from 19.59% null model to 6.9% in the final model (model IV). The Proportional Change in Variance (PCV) also increases from 20.01% from model II to 44.64% in model IV (a model adjusted for individual and community level variables), which indicates 44.64% of the community variance observed in the null model was explained by both individual and community-level factors in full model (model IV). Besides, model fitness was checked using deviance and the model with the lowest deviance (model IV) was the best-fitted model. The variance inflation factor (VIF) is 1.39 which is lower than the recommended cut-off point indicating the absence of a significant correlation between independent variables (Table 3).

**Table 3:** Random effects, model fitness, and multicollinearity

Random effects	Model I (null)	Model II	Model III	Model IV
Community level variance (SE)	0.56 (0.13)	0.45(0.12)	0. 31 (0.07)	0.31 (0.07)
ICC (%)	14.59	14.34	8.80	6.90
PCV (%)	1.00	20.01	44.64	44.64
Model fitness				
LLR (Log-likelihood Ratio)	−10,133.49	−10,107.14	−10,123.44	−10,097.17
Deviance (−2LL)	20,266.98	20,214.28	20,246.88	20,194.34
Multicollinearity				
VIF	–	–	–	1.39

*ICC* intracluster correlation coefficient, *PCV* proportional change in variance, *1.00* reference, *VIF* variance inflation factor

### Factors associated with unintended pregnancy

In multilevel logistic regression analysis, marital status, educational status, sex of household head, and knowledge of contraceptive method from individual-level variables and sub-Saharan African region from community-level factors were significantly associated with unintended pregnancy.

The odds of unintended pregnancy was decreased by 10% (AOR = 0.90, 95%CI 0.59–0.95) and 14% (AOR = 0.86 95%CI 0.75–0.98) among adolescent girls and young women who know traditional and modern contraceptive methods respectively as compared to women who didn’t know any method. Married women had 20% (AOR = 0.80, 95%CI 0.73–0.88) decreased odds of unintended pregnancy as compared to single women.

The likelihood of unintended pregnancy among adolescent girls and young women from female-headed households was decreased by 14% (AOR = 0.86,95%CI 0.78–0.94) as compared to male-headed households. Adolescent girls and young women with higher educational levels had 29% (AOR = 0.71 95%CI 0.52–0.97) decreased odds of unintended pregnancy as compared to women with no formal education.

Looking at community-level variables, adolescent girls and young women from Central Africa, southern Africa, and Eastern Africa had 2.09 (AOR = 2.09,95%CI 1.23–3.55), 5.23 (AOR = 5.23, 95%CI 2.71–10.09) and 1.07 (AOR = 1.07,95%CI 1.07–2.66) respectively times higher odds of unintended pregnancy as compared to women in Western Africa (Table 4).

**Table 4:** Multilevel analysis of determinants of unintended pregnancy in sub-Saharan Africa 2006–2019

Variables	Model I (Null)	Model II AOR 95%CI	Model III AOR 95%CI	Model IV AOR 95%CI
Individual-level variables				
Age				
15–19	–	1.00	–	1.00
20–24	–	0.96(0.89 -1.03)	–	0.96(0.89–1.03)
Educational level				
No education	–	1.00	–	1.00
Primary	–	1.04(0.94–1.14)	–	1.86(0.97–1.15)
Secondary	–	1.08(0.91–1.20)	–	1.07(0.97–1.18)
Higher	–	0.72(0.52–0.99)	–	0.71(0.52–0.97)^a^
Sex of household head				
Male		1.00		1.00
Female		0.85(0.78–0.94)		0.86(0.78–0.94)^c^
Marital status				
Single	–	1.00	–	1.00
Married	–	0.84(0.72, 0.99)	–	0.80(0.73–0.88)^c^
Wealth status				
Poorest	–	1.00	–	1:00
Poorer	–	1.05(0.95–1.16)	–	1.05(0.95–1.16)
Middle	–	1.01(0.91–1.13)	–	1.02(0.92–1.13)
Richer	–	1.03(0.92–1.15)	–	1.04(0.93–1.17)
Richest	–	1.10(0.97–1.25)	–	1.12(0.98–1.29)
Media exposure				
No	–	1.00	–	1.00
Yes	–	1.06(0.98–1.15)	–	1.06(0.98–1.15)
Knowledge of contraceptive				
No	–	1.00	–	1.00
Traditional	–	0.91(0.51–1.41)	–	0.90(0.59–0.95)^b^
modern		0.86(0.75–0.98)		0.86(0.75–0.98)^b^
Smoking				
No	–	1.00	–	1.00
Yes	–	1.03(0.89–1.20)	–	1.03(0.99–1.08)
Distance from health facility				
Not a big problem	–	0.97(0.90–1.05)	–	0.97(0.91–1.05)
Big problem	–	1.00	–	1.00
Heard family planning from media				
No		1.00	–	1.00
yes		0.97(0.91–1.05)	–	0.97(0.90–1.05)
Covered by health insurance				
No		1.00	–	1.00
Yes		0.91(0.80–1.04)	–	0.91(0.80–1.04)
Occupation				
Not working		1.00	–	1.00
working		0.98(0.91–1.05)	–	0.98(0.91–1.05)
Community-level variables				
Residence				
Urban	–	–	1.00	1.00
Rural	–	–	0.98(0.91–1.05)	1.02(0.94–1.12)
SSA Region				
Central Africa	–	–	2.09(1.23–3.56)	2.09(1.23–3.55)^c^
Southern Africa	–	–	5.18(2.69–9.99)	5.23(2.71–10.09)^c^
East Africa	–	–	1.68(1.07–2.64)	1.07(1.07–2.66)^b^
West Africa	–		1.00	1.00

^a^significant at alpha <= 0.05

^b^significant at alpha<= 0.01

^c^significant at alpha<= 0.001

## Discussion

This study investigated the prevalence and correlates of unintended pregnancy among adolescent girls and young women in sub-Saharan Africa with data from the most recent Demographic and Health Surveys of 36 Sub-Saharan countries. The estimated prevalence of unintended pregnancy was 30.10% with a 95% CI (29.38–30.74). This estimate is higher than the previous pieces of evidence in India (16.9%) [32], Nepal (22.7%) [19], Sri Lanka (17.2%) [16], and South Asian countries (19.1%) [18]. However, it is lower than the prevalence in Pakistan (38.2%) [15], Kenya (41%) [21], and Uganda (37%) [22]. This difference could be attributed to the variation in intervention to reduce the unmet need for contraception, and unintended pregnancies among women which are critical components of family planning programs in developing countries [33].

The odds of untended pregnancy among adolescent girls and young women who know contraceptive methods (traditional and modern) decreased as compared to those who didn’t know any methods. This finding is in line with studies in Pakistan [15] and Sri Lanka [16]. Besides, Studies in Bangladesh and, Nepal reported that poor contraceptive knowledge was significantly associated with unplanned pregnancy [34, 35]. Since contraceptive awareness is directly related to its utilization [36–38], women with knowledge of any contraceptive method may have better utilization of family planning methods which help to avoid unintended pregnancy. Thus, it is essential to implement initiatives to improve community knowledge about contraceptive methods with a primary focus on adolescent girls and young women.

This study revealed that education was an important predictor of unintended pregnancy where the odds of unintended pregnancy was decreased among women with higher education. This finding is consistent with a study in Ghana [13, 14], Pakistan [15], and Sri Lanka [16]. The documented pieces of evidence indicated that literate women have a better understanding of their rights and responsibilities and have more freedom, control, and participation in decisions primarily focusing on contraception use and family planning [39–42]. On the contrary, a study in Nigeria found that better-educated women had higher odds of reported unplanned pregnancies [43].

Consistent with previous pieces of literature [16, 18, 20] married women had lower odds of unintended pregnancy as compared to single ones. This may reflect that a more stable relationship between the couples might have encouraged them to have better decision-making and utilization of family planning methods and timing of conception [44].

Similarly, the odds of unintended pregnancy among adolescent girls and young women from female-headed households was lower as compared to those of male-headed households. This might be due to shared family planning decisions in those female-headed households [20] since the women’s capacity of making reproductive health decisions has a significant effect on unintended pregnancy in the previous study [14]. Besides, the evidence shows that husband-only or wife-only decision-making is associated with a higher risk for women in having both unmet needs for contraception and unintended pregnancy [45]. The region was also a significant predictor of unintended pregnancy in sub-Saharan Africa. Those adolescent girls and young women from central, southern, and eastern Africa had higher odds of unintended pregnancy as compared to those from the western region. This might have happened due to the geographic variation of reproductive health services like contraceptive practice in the region since SSA’s family planning situation remains challenged by weak health systems [46]. For instance, in eastern and southern Africa adolescent sexual and reproductive health services require much promotion to ensure explicit understanding in the community by overcoming diverse cultural confrontations [47]. On the other hand, a recent study has shown that adolescent health services including family planning are increasingly available in West Africa [48].

In general, a closer look at sub-Saharan Africa shows how far behind African countries are in preventing these unplanned pregnancies. The relationship between social and economic development and unintended pregnancy goes both ways. When women and girls are not empowered or lack autonomy, they are less likely to use contraceptives, and then they are prone to unintended pregnancy.

This study has strengths and limitations. It is a pooled data analysis that yields a high sample size and then high power. The other strength is the study considers the clustering effect by applying an advanced model to get reliable standard error and estimates. However, it has the following limitations. Due to the cross-sectional study design, it is difficult to establish the temporal relationship between the predictors and the outcome variable. Besides, there could be a possibility of social desirability bias when women report whether their pregnancy was intended or not.

## Conclusion

Unintended pregnancy is a public health problem in sub-Saharan Africa. Educational status, knowledge of contraceptive methods, marital status, sex of household head, and sub-Saharan African region had a significant statistical association with unintended pregnancy.

Therefore, focusing on educating adolescent girls and young women, and improving their knowledge about family planning services through youth-friendly services is vital to reduce unintended pregnancy in sub-Saharan Africa. Moreover, the findings in this study have valuable input to policymakers to reframe their focus on strengthening women’s empowerment in decision-making through education, and in providing youth-friendly services for adolescent girls and young women. In general, the government of countries in sub-Saharan Africa and other global and local stakeholders should work hard on achieving target 3.7 of the Sustainable Development Goals (SDGs) which states: “by 2030, to ensure universal access to sexual and reproductive health-care services, including for family planning, information and education, and the integration of reproductive health into national strategies and programs” [49].

## Data Availability

Data is available online and it is possible to access it from www.measuredhs.com.

## Abbreviations

AOR: Adjusted Odds Ratio
DHS: Demographic health survey
CI: Confidence Interval
EAs: Enumeration areas
ICC: Intra-cluster Correlation
LLR: Log likelihood ratio
LR: Likelihood ratio
SSA: Sub-Saharan Africa
